# Short-term safety results from compassionate use of risdiplam in patients with spinal muscular atrophy in Germany

**DOI:** 10.1186/s13023-022-02420-8

**Published:** 2022-07-19

**Authors:** Andreas Hahn, René Günther, Albert Ludolph, Oliver Schwartz, Regina Trollmann, Patrick Weydt, Markus Weiler, Kathrin Neuland, Martin Sebastian Schwaderer, Tim Hagenacker, the Risdiplam Compassionate Use Program Group

**Affiliations:** 1 Department of Child Neurology, University of Gießen, Giessen, Germany; 2 Department of Neurology, University Hospital Carl Gustav Carus, Technische Universität Dresden, Dresden, Germany; 3 Department of Neurology, University of Ulm, Ulm, Germany; 4 Department of Pediatric Neurology, Münster University Hospital, Münster, Germany; 5 Division of Pediatric Neurology, Department of Pediatrics, Friedrich-Alexander-Universität Erlangen-Nürnberg, Erlangen, Germany; 6 Department of Neurodegenerative Diseases and Gerontopsychiatry/Neurology, University of Bonn Medical Center, Bonn, Germany; 7 Department of Neurology, Heidelberg University Hospital, Heidelberg, Germany; 8 Roche Pharma AG, Grenzach-Wyhlen, Germany; 9 Department of Neurology, University Hospital Essen, Essen, Germany

**Keywords:** Spinal muscular atrophy, Risdiplam, Splicing modifier, Survival of motor neuron 1 protein, Compassionate use, Real-world data

## Abstract

**Background:**

The oral, selective *SMN2*-splicing modifier risdiplam obtained European approval in March 2021 for the treatment of patients ≥ 2 months old with a clinical diagnosis of 5q-associated spinal muscular atrophy (SMA) 1/2/3 or with 1–4 *SMN2* gene copies. For the preceding 12 months, this compassionate use program (CUP) made risdiplam available to patients with SMA1/2 in Germany who could not receive any approved SMA therapy.

**Patients and methods:**

Patients with SMA1/2, aged ≥ 2 months at enrollment, could be included if they were not eligible for, no longer responsive to, or not able to tolerate nusinersen or not able to receive onasemnogene abeparvovec. Oral risdiplam dosing ranged from 0.2 mg/kg to 5 mg depending on age and weight. All treatment decisions were made by the attending physicians, who were required to report all adverse events (AEs).

**Results:**

Between March 12, 2020 and March 30, 2021, 36 patients with SMA1 and 98 patients with SMA2 were enrolled, with 31 patients and 80 patients receiving ≥ 1 risdiplam dose, respectively. The median (range) age was 10.5 (3–52) years in the SMA1 cohort, and 26.5 (3–60) years in the SMA2 cohort. 22.2% of patients with SMA1 and 48.0% with SMA2 were treatment-naïve. Most patients were not eligible/could not continue to receive nusinersen due to scoliosis/safety risk (SMA1: 75.0%; SMA2: 96.9%), risks associated with sedation (77.8%; 63.3%), or loss of efficacy (30.6%; 12.2%). Safety data were generally in line with the safety profile of risdiplam in ongoing clinical studies. Gastrointestinal disorders were the most common AEs. For patients with SMA1, 30 AEs were reported in 13 cases with 2 serious AEs in 1 patient. For SMA2, 100 AEs were documented in 31 case reports, including 8 serious AEs in 2 patients.

**Conclusions:**

We present the first real-world safety data of risdiplam in patients with SMA in Germany. Our observations indicated no new safety signals under real-world conditions. Real-world SMA1/2 populations comprise considerable numbers of patients who are not eligible for gene therapy and cannot tolerate or have failed nusinersen treatment. This medical need may be addressed by oral risdiplam.

**Supplementary Information:**

The online version contains supplementary material available at 10.1186/s13023-022-02420-8.

## Introduction

5q-associated spinal muscular atrophy (SMA) is a severe, progressive neuromuscular disease leading to devastating muscle atrophy [1]. The functional loss of the *SMN1* gene—which encodes the SMN (survival motor neuron) protein—causes degeneration of spinal cord motor neurons. Humans have several copies of a paralogous adjacent gene named *SMN2,* which has a slightly altered sequence resulting in imperfect splicing—with skipping of exon 7 [2]. Only approximately 10% of *SMN2*-derived transcripts produce functional SMN protein [2, 3], thus the *SMN2* gene cannot compensate for biallelic *SMN1* mutations. However, disease severity inversely correlates with *SMN2* copy number [4, 5].

The phenotypic spectrum of SMA is reflected in the classification of the disease (SMA0–4) [1]. Infants with the very severe type SMA0 develop symptoms in utero and die within 6 months after birth. Children with SMA1 have feeding and respiratory problems before 6 months of age and usually develop respiratory failure, the main cause of death, by the age of 2 years. Patients with SMA2 learn to sit independently but will never be able to walk without assistance. Although life expectancy is reduced due to respiratory and/or bulbar dysfunction, most patients survive to adulthood. Patients with SMA3 and SMA4 have a normal life expectancy. They develop milder symptoms later in life; in SMA3 muscle function progressively declines in the second decade of life, which decreases walking ability and other motor functions.

The current standard therapies for SMA include the anti-sense oligonucleotide (ASO) nusinersen (*Spinraza*, Biogen Netherlands B.V., Badhoevedorp, the Netherlands), which is administered by intrathecal injection and indicated for treatment of all patients with SMA [6], and the gene transfer therapy onasemnogene abeparvovec (*Zolgensma*, Novartis Gene Therapies EU Limited, Dublin, Ireland), which was approved by the European Medicines Agency (EMA) for patients with diagnosed SMA1 and/or with 1–3 copies of the *SMN2* gene [7].

Recently, the oral, selective *SMN2*-splicing modifier risdiplam (*Evrysdi*, Roche Pharma, Grenzach-Wyhlen, Germany) has been approved [8]. Risdiplam was designed to promote inclusion of exon 7 in the *SMN2* mRNA transcript, thereby increasing the production of functional SMN protein. The ongoing Phase 3 parts of the risdiplam studies FIREFISH (SMA1) and SUNFISH (SMA2/3) both met their primary efficacy endpoints after 12 months [9, 10], with 93% of patients with SMA1 surviving after 24 months [11](see Additional file 1: Table S1).

The US Food and Drug Administration (FDA) approved risdiplam for patients 2 months of age and older with SMA in August 2020. Risdiplam was approved by the EMA on March 26, 2021, for the treatment of patients with SMA, an age of 2 months and older, and a clinical diagnosis of SMA1, 2 or 3 or with 1–4 *SMN2* gene copies [8]. Before this, German patients who were not eligible to receive, were no longer responsive to, or not able to tolerate nusinersen and/or onasemnogene abeparvovec had no available therapy options, since they did not have access to clinical trials with risdiplam after January 2020. To bridge this treatment gap, this compassionate use program (CUP) provided risdiplam to these patients with SMA1/2. In a global effort, Roche has conducted similar national CUPs in more than 50 countries, providing pre-approval risdiplam access to more than 2000 patients (Roche Pharma AG, data on file, status 2021).

Here, we report the first real-world data on patient characteristics and short-term safety for risdiplam in clinical practice in Germany.

## Methods

According to German regulations (Arzneimittelhärtefallverordnung, AMHV), CUPs may provide an investigational drug to patients with serious or life-threatening conditions who cannot be treated with an approved therapy; the CUP is terminated as soon as the therapy becomes available on the market.

This CUP with risdiplam started on March 12, 2020, following a written confirmation of a valid notification issued by the responsible Health Authority (Bundesinstitut für Arzneimittel und Medizinprodukte, BfArM); the CUP was terminated on March 30, 2021 following EMA approval, and data cutoff for reportings was June 14, 2021. Thus this analysis covers reporting collected over 15 months. The CUP was first limited to patients with SMA1 and was extended to SMA2 in July 2020, upon filing the risdiplam marketing authorization application with the EMA. Expected participant numbers were initially ~ 10 patients with SMA1 and 100–250 patients with SMA2; but for SMA1, expected patient numbers were increased several times and last amended to 45 patients on March 8, 2021. All patients and/or legally authorized representatives provided written informed consent to participate in the CUP.

### Patients

Patients were eligible for this CUP if they were ≥ 2 months old at enrollment and had a confirmed diagnosis of 5q-associated SMA1 or SMA2 (including genetic confirmation of homozygous deletion or compound heterozygosity predictive of loss of function of the *SMN1* gene). Participants had to be non-eligible for, no longer responsive to or not able to tolerate any approved treatment options. Reasons for this included a medical condition (e.g. complex spinal abnormality such as severe scoliosis or vertebral fusion and extensive instrumentation, high risks associated with sedation that precluded intrathecal administration of nusinersen at an acceptable safety risk, evident loss of efficacy assessed by the treating physician as documented by a clinically meaningful decline on a motor scale commonly used to monitor SMA (e.g. CHOP-INTEND (Children's Hospital of Philadelphia Infant Test of Neuromuscular Disorders), HFMSE (Hammersmith Functional Motor Scale Expanded), RULM (Revised Upper Limb Module), MFM (Motor Function Measure)), and/or a contraindication listed in the prescribing information (e.g. hypersensitivity against nusinersen/onasemnogene abeparvovec or their respective excipients). In addition, female patients of childbearing potential had to have a negative pregnancy test and consented to using highly effective contraception during risdiplam therapy and for at least 1 month after the last dose; males of reproductive age consented to using highly effective contraception during and at least 4 months after risdiplam treatment. At the time of the CUP, patients could not receive risdiplam in any ongoing clinical trial.

Patients were excluded if they had any serious medical condition or treatment that, in the treating physician’s judgment, precluded the patient’s safe participation. There had to be a minimal treatment-free period of 120 days before starting risdiplam therapy following the administration of nusinersen, or of ≥ 12 weeks following onasemnogene abeparvovec therapy. Additional inclusion criteria were normal liver function tests, coagulation parameters, platelets and troponin-I at 12 weeks after administration of onasemnogene abeparvovec or at least 1 month after tapering off corticosteroids, whichever came last.

### Treatment and dosing

The decision for risdiplam therapy was made by the attending physician based on a positive benefit/risk assessment. All assessments and treatment decisions were at the discretion of the treating physician and in accordance with applicable global and local regulatory requirements, including the documentation and reporting of relevant safety data. Participating physicians were qualified (pediatric) neurologists with adequate experience in SMA treatment, sufficient knowledge to administer the unauthorized medicinal product, and access to a pharmacy with adequate facilities and qualified personnel to reconstitute the risdiplam oral solution. Physicians were advised to perform a complete medical history and physical exam prior to initiating therapy.

Risdiplam could be self-administered by the patient or a parent/caregiver at home. At the first visit, participants were instructed to orally administer risdiplam (0.75 mg/mL aqueous solution) once daily at approximately the same time each day. Twice-monthly visits were recommended to recalculate the dose based on bodyweight and resupply patients with medication. The dosing scheme was identical to the dose recommendation in the *Evrysdi* Summary of Product Characteristics [8]: 0.2 mg/kg risdiplam for patients between 2 months and 2 years of age, 0.25 mg/kg for patients > 2 years of age and < 20 kg, and a fixed dose of 5 mg for patients > 2 years of age and ≥ 20 kg.

Treatment with risdiplam was continued as long as the treating physician considered treatment beneficial for the patient. Pre-defined discontinuation criteria included any medical condition that could jeopardize the patient’s safety with continued treatment, non-compliance, patient/caregiver request and unacceptable toxicity (at the discretion of the treating physician). Coadministration of risdiplam with flavin monooxidase modulators (e.g., methimazole), and octamer-binding protein 2 and multidrug and toxin extruder substrates was to be avoided.

### Adverse event documentation

The adverse event (AE) severity was assessed by the NCI CTCAE v5.0 (National Cancer Institute Common Terminology Criteria for Adverse Events) grading scale. The treating physicians were required to report all AEs, serious AEs (SAEs), special situation reports and product complaints until 28 days after the final dose the patient received in the CUP. Following the termination of the program, final inquiries to the treating physicians were made to check that all AE/SAE reports were received properly and to correct the reports accordingly if necessary. This took place until June 14, 2021.

### Statistics

Data on patient characteristics at baseline, including sex, age, previous therapies (treatment duration and reason why patients were not eligible or could not continue to receive an approved therapy), motor function (age at onset and highest motor function milestone achieved at baseline) were recorded. According to legal regulations for compassionate use, no effectiveness data were systematically collected. AEs/SAEs were classified by Medical Dictionary for Regulatory Activities (MedDRA) System Organ Class (SOC) and Preferred Term (PT). Percentages of AEs and SAEs were calculated based on the total number of AEs.

The patient collective was analyzed using standard descriptive methods. For continuous variables, the median and corresponding minimum and maximum values were reported. For categorical variables, the absolute and relative frequencies were reported. Analyses were conducted with R Version 3.6.1 (R Foundation for Statistical Computing, Vienna, Austria) and PAIRS (Gandysoft, Henderson, Nevada, USA).

## Results

### Patient exposure

**Fig. 1:**
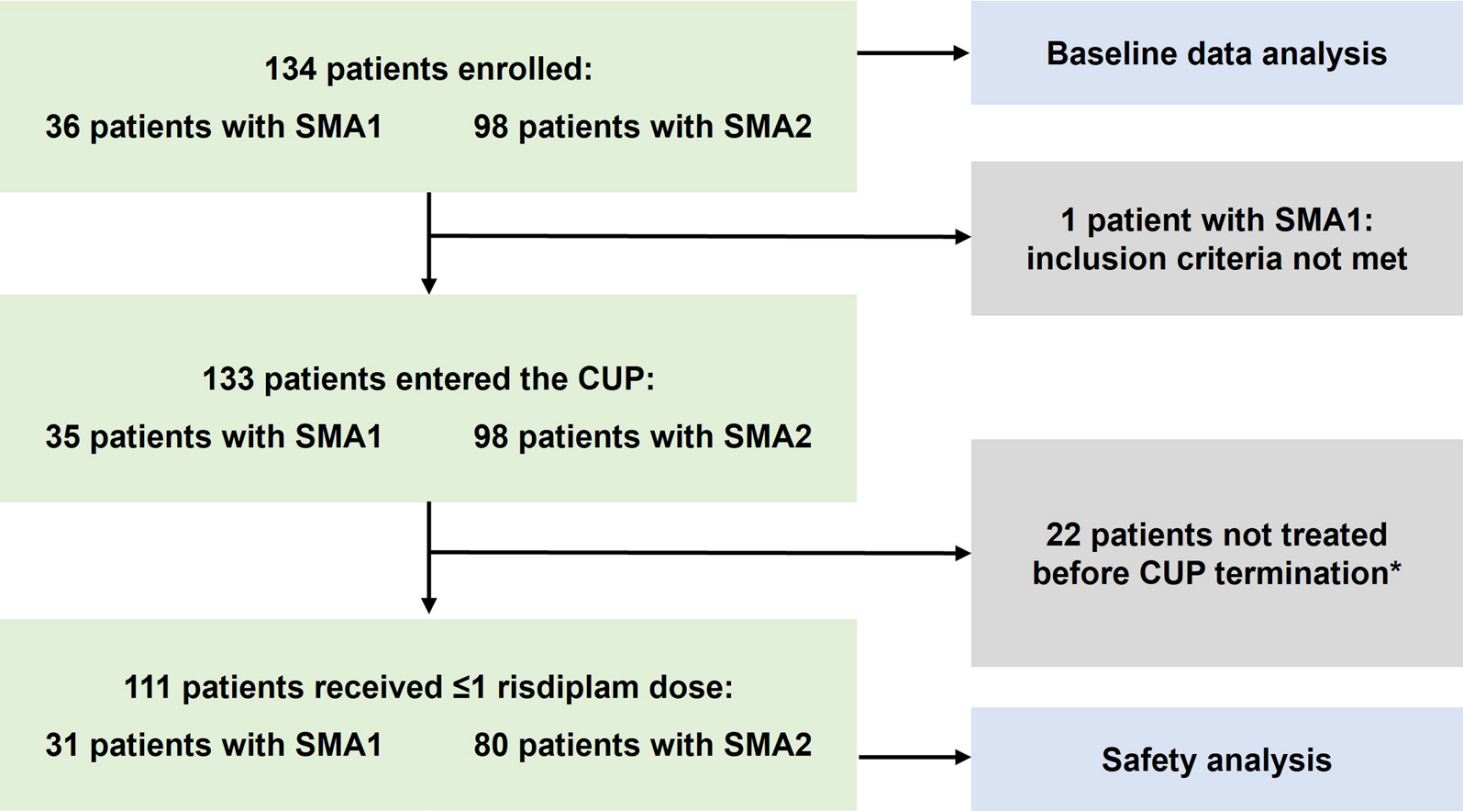
Patient disposition. Numbers of patients in the compassionate use program on which the baseline data analysis and safety analysis were based. *22 patients had not yet passed the 120-day wash-out period for previous nusinersen treatment before the CUP was terminated

Between March 12, 2020, and March 30, 2021, 36 patients with SMA1 and 98 patients with SMA2 were enrolled at 23 centers, including pediatric and neurologic departments. Patient disposition is shown in Fig. 1. The first patients started risdiplam treatment on June 26, 2020 (SMA1), and on September 14, 2020 (SMA2). Mean ± SD (standard deviation) duration of risdiplam treatment was 5.0 ± 2.7 months in the SMA1 cohort and 3.4 ± 1.9 months in the SMA2 cohort.

### Patient characteristics

**Fig. 2:**
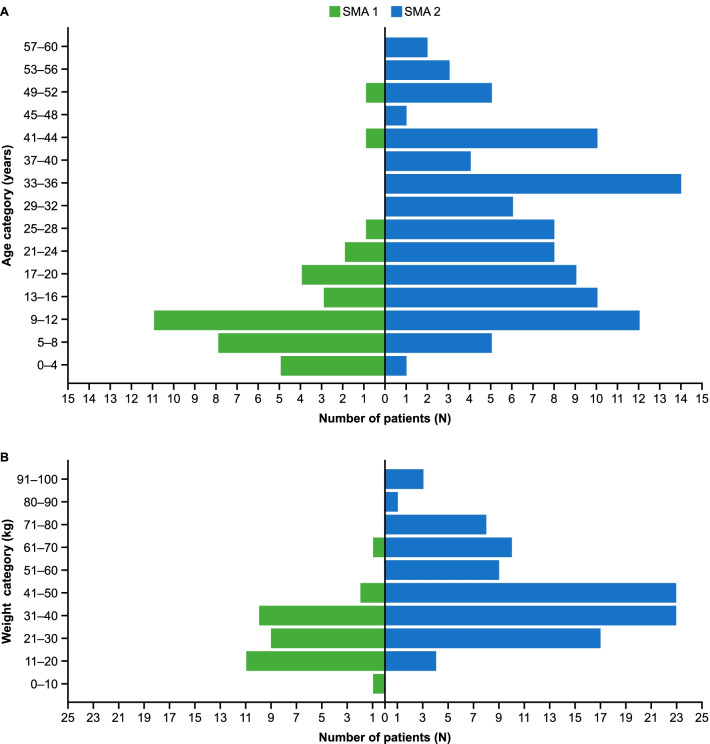
Age (**A**) and bodyweight (**B**) distribution. Number of patients with SMA1 (green bars, n = 36) and SMA2 (blue bars, n = 98) in age (**A**) and bodyweight (**B**) categories at baseline in the compassionate use program. SMA1, spinal muscular atrophy type 1; SMA2, spinal muscular atrophy type 2

Baseline demographic characteristics, baseline motor function and pre-treatment characteristics both of the enrolled population and patients who were treated with risdiplam are presented in Table 1. The distribution of patients by age and bodyweight is shown in Fig. 2: 52.8% of patients with SMA1 were 5–12 years old, with a range of 3–52 years, while the SMA2 population included mostly adults across all age groups (range 3–60 years).

**Table 1:** Baseline demographic characteristics, baseline motor function and pre-treatment characteristics

Baseline characteristics	Enrolled patients		Patients treated with risdiplam	
	SMA1	SMA2	SMA1	SMA2
Total number of patients	36	98	31	80
Female/male, n (%)	16/20 (44.4/55.6)	59/39 (60.2/39.8)	14/17 (45.2/54.8)	49/31 (61.3/38.8)
Mean ± SD age at start of enrollment, years	13.1 ± 10.4	27.6 ± 14.1	13.5 ± 11.1	29.0 ± 14.0
Mean ± SD age at SMA onset, years	2.8 ± 2.3	11.3 ± 4.1	2.65 ± 2.18	11.1 ± 4.4
Mean ± SD weight, kg	27.3 ± 11.2*	46.4 ± 19.0	25.8 ± 9.84*	46.3 ± 19.4
Prior therapies				
Nusinersen, n (%)	28 (77.8)	51 (52.0)	23 (74.2)	37 (46.3)
Mean ± SD nusinersen treatment duration, months	31.9 ± 16.6	25.7 ± 11.2*	28.6 ± 15.7	23.9 ± 11.3
Onasemnogene abeparvovec, n (%)	0	0	0	0
No SMA pre-treatment, n (%)	8 (22.2)	47 (48.0)	8 (25.8)	43 (53.8)
Baseline motor function, n (%) Current level of function/highest motor function achieved				
Supports head unaided	6 (16.7)/10 (27.8)	33 (33.7)/7 (7.1)	5 (16.1)/7 (22.6)	22 (27.5)/5 (6.3)
Sitting unaided	0/0	41 (41.8)/66 (67.3)	0/0	36 (45.0)/53 (66.3)
Crawls on stomach	0/0	3 (3.1)/17 (24.4)	0/0	3 (3.8)/15 (18.8)
Stands unaided	0/0	0/7 (7.1)	0/0	0/6 (7.5)
Walks unaided	0/0	0/0	0/0	0/0
None of the above	30 (83.3)/26 (72.2)	21 (21.4)/1 (1.0)	26 (83.9)/24 (77.4)	19 (23.8)/1 (1.3)

*2 patients with missing data

*SD* standard deviation, *SMA1* spinal muscular atrophy type 1, *SMA2* spinal muscular atrophy type 2

**Fig. 3:**
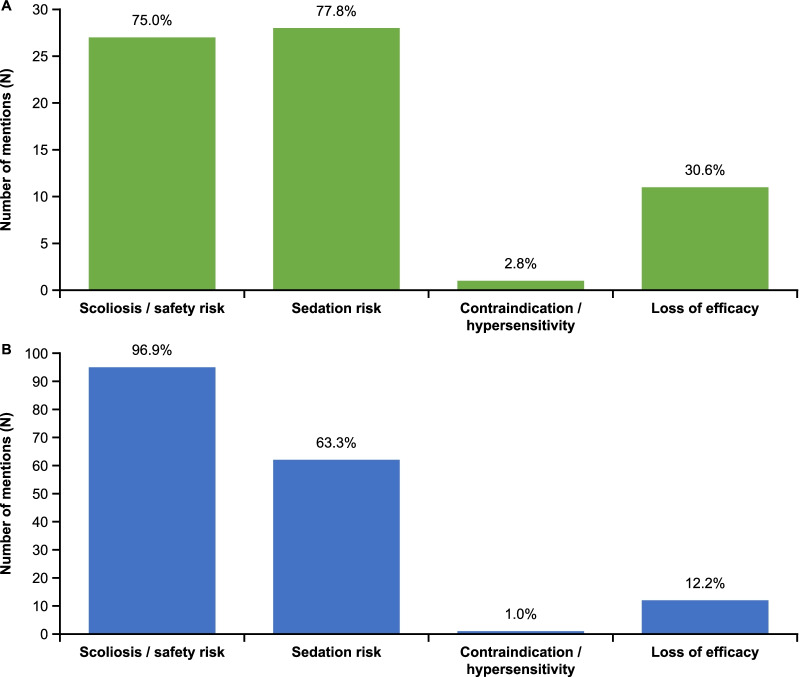
Reasons precluding nusinersen use. Reasons why patients with **A** SMA1 (green bars, n = 36) and **B** SMA2 (blue bars, n = 98) were deemed by their treating physician as not eligible or not able to continue to receive nusinersen at baseline. Multiple answers were possible. SMA1, spinal muscular atrophy type 1; SMA2, spinal muscular atrophy type 2

According to the questionnaire data collected at baseline, for both SMA1/2 the dominant reasons why patients were deemed by their physician to be not eligible/not able to continue to receive nusinersen were issues related to its intrathecal administration (Fig. 3, multiple answers were possible).

Four patients with SMA1 were included before onasemnogene abeparvovec became available in Germany approximately 2 months after the CUP started. For the remaining 32 patients with SMA1 and all patients with SMA2, the documented medical reason for onasemnogene abeparvovec ineligibility was an unacceptable safety risk.

### Adverse events

#### Overview

During the safety reporting period, a total of 130 AEs were observed in 44 case reports. For SMA1, 30 AEs were reported in 13 cases with 2 SAEs in 1 patient. For SMA2, 100 AEs were reported in 31 cases, including 8 SAEs in 2 patients.

#### SAEs

An overview over SAEs by MedDRA SOC is presented in Table 2, and by MedDRA PT in Additional file 1: Table S2. No SAEs with a fatal outcome were reported in this CUP. Risdiplam treatment was permanently discontinued at the patient's request in a single patient with SMA2, who suffered from SAEs with pre-existing diverticulitis, which exacerbated 3 times during therapy. For the other 2 patients who experienced SAEs, no information about any alteration or discontinuation of risdiplam treatment was documented.

**Table 2:** List of SAEs by MedDRA system organ class

Serious adverse events, n (%)*	SMA1	SMA2	Total
Total number	2 (6.7)	8 (8.0)	10 (7.7)
Gastrointestinal disorders	–	5 (5.0)	5 (3.8)
General disorders and administration site conditions	–	1 (1.0)	1 (0.8)
Infections and infestations	2 (6.7)	2 (2.0)	4 (3.0)

Of 111 patients who received at least one dose of risdiplam (31 patients with SMA1 and 80 patients with SMA2), 3 patients (1 patient with SMA1 and 2 patients with SMA2) experienced at least 1 SAE

*Percentages are based on total number of AEs (30 in SMA1 and 100 in SMA2). MedDRA, Medical Dictionary for Regulatory Activities; SAE, serious adverse event; SMA1, spinal muscular atrophy type 1; SMA2, spinal muscular atrophy type 2

Among the 3 patients with SAEs, one pre-school child with SMA1 developed pneumonia twice during the reporting period. The patient recovered following hospitalizations and antibiotic therapy. No causality with risdiplam for the first pneumonia was reported, whereas the second pneumonia was reported as related to risdiplam.

Approximately 2 weeks after the start of risdiplam, an adult patient with SMA2 and pre-existing diverticulitis, deep vein thrombosis and pain, presented with fever, abdominal pain, two episodes of diarrhea, constipation and increased C-reactive protein levels. An abdominal computer tomography showed a segmented sigma diverticulitis with occult perforation and inflamed inhibition of the surrounding peritoneal lipid tissue, with involvement of a neighboring ileum fold. The patient recovered after hospitalization and antibiotic therapy. A second episode of acute diverticulitis responded well to out-patient antibiotic treatment. Risdiplam therapy was not altered during both these episodes, but paused repeatedly to resolve diarrhea. A third sigmoid diverticulitis exacerbation resolved rapidly after discontinuation of risdiplam. Due to 3 episodes of acute diverticulitis, risdiplam was permanently discontinued at the patient's request. The treating physician assessed the sigmoid perforation, both episodes of worsening of diverticulitis, fever, abdominal pain and constipation as not related to risdiplam, and sigmoid diverticulitis as unknown causality with risdiplam.

In a preadolescent child with SMA2 initially treated with nusinersen, serious esophageal hypomotility was observed during risdiplam treatment approximately 2 weeks after starting risdiplam therapy, which persisted at the time of reporting: The patient was not able to eat anything, and an imaging procedure showed that the esophagus was too sluggish. There was no documentation regarding a causal relationship with risdiplam treatment.

#### Non-serious AEs

**Fig. 4:**
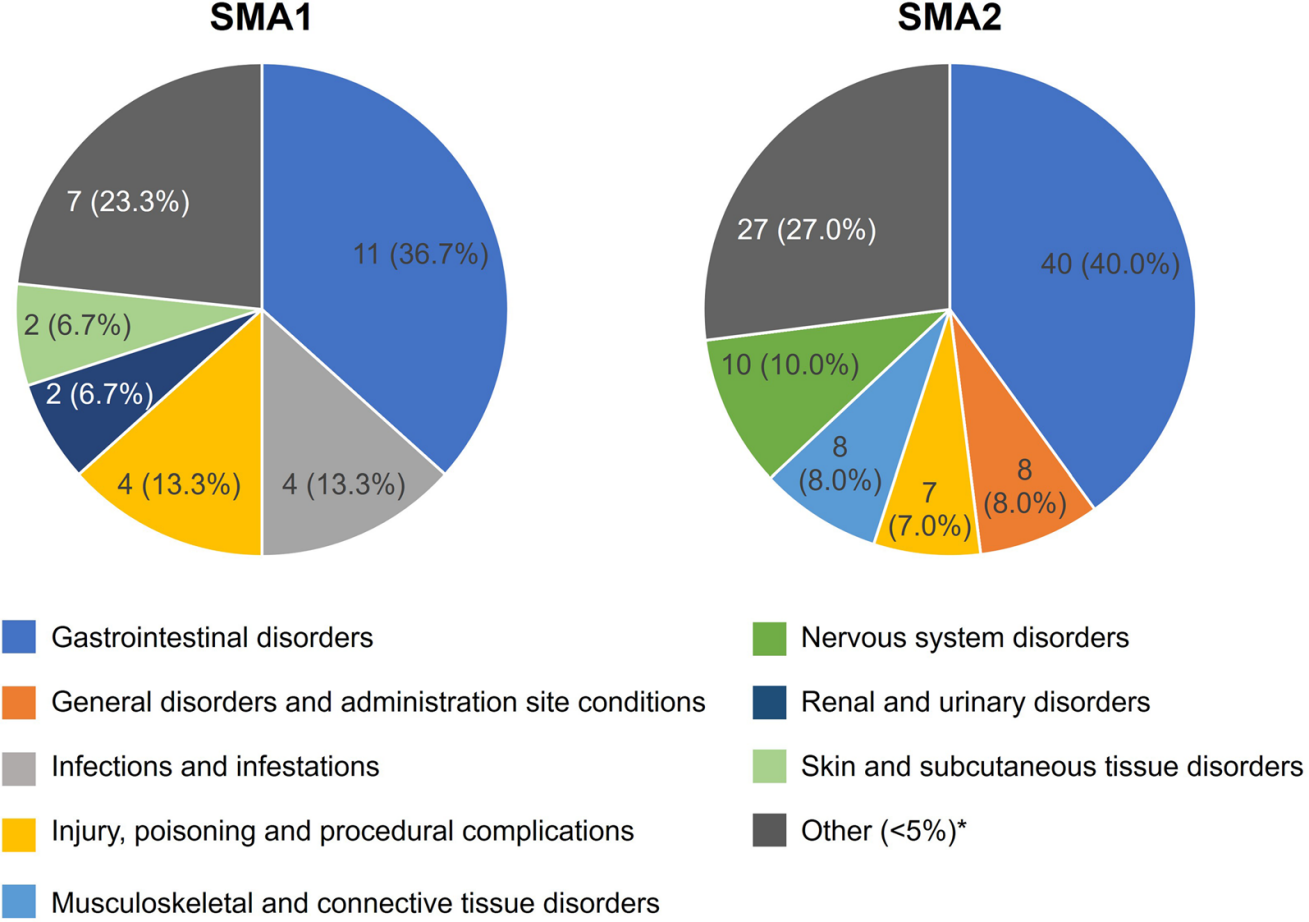
AEs by MedDRA System Organ Class. Safety analysis was based on 111 patients who received at least one dose of risdiplam (31 patients with SMA1 and 80 patients with SMA2). Percentages are based on the total number of events (30 and 100 AEs in SMA 1 and SMA2, respectively). AE, adverse event; MedDRA, Medical Dictionary for Regulatory Activities; SMA1, spinal muscular atrophy type 1; SMA2, spinal muscular atrophy type 2

The most common non-serious AEs in SMA1 were gastrointestinal disorders: diarrhea was observed 3 times (10.0% of all AEs), abdominal pain and salivary hypersecretion both occurred 2 times (6.7% of all AEs). In SMA2, diarrhea was the most common non-serious AE (9 events, 9.0% of all AEs), followed by headache (6 events, 6.0% of all AEs), and aphthous ulcer, constipation, nausea and circumstance or information capable of leading to medication error (all 4 events, 4.0% of all AEs). Figure 4 presents an overview of all AEs shown as MedDRA SOC categories. A listing of all non-serious AEs by MedDRA SOC and PT is presented in Additional file 1: Table S3.

## Discussion

Although this CUP was restricted to patients with SMA1 and SMA2, considerably more and older patients than was initially expected were included into the program, which underlined an unmet medical need for an additional treatment option.

Baseline demographic characteristics and motor function of the patient population were generally characteristic of patients with SMA1 and SMA2. The large proportion of adults and older children in the SMA1 population can be explained by advances in respiratory and nutritional care that have extended survival considerably [12, 13]. Increasingly applied supportive nutritional and respiratory measures in industrialized countries also have reduced mortality in SMA1 to approximately 30% at the age of 2 years [14]. However, before approval of the first genetic SMA therapies, nusinersen in 2017 and onasemnogene abeparvovec in 2020, which substantially increased survival [15], the median life expectancy for SMA1 was estimated at < 2 years of age [1].

German patients have access to receiving these genetic therapies in accordance with the label. There are sufficient capacities for intrathecal injections in qualified and specialized centers, and the costs are covered by the German healthcare system. However, more than a fifth of patients with SMA1 and nearly half of patients with SMA2 in this CUP did not receive any prior SMA treatment.

Among pre-treated patients with SMA1/2, primarily administration-related complications precluded them from receiving nusinersen: This reflects that scoliosis, previous spinal surgeries such as spondylodesis and joint contractures are common in patients with SMA. Despite significant progress from conventional lumbar injection (such as imaging-guided procedures, alternative delivery routes and catheter systems), recurrent intrathecal administration of nusinersen over many years remains challenging [16–18]. Similarly, respiratory difficulties and anatomical conditions complicating intubation may result in unacceptably high risks associated with anesthesia [19]. Respiratory risk can be high for both tracheostomized infants with SMA1 and less breathing-impaired patients with SMA2, who have no tracheostoma and are thus more difficult to treat for sedation-induced oxygen deficiency. Furthermore, Phase 3 study data [20] and real-world data [21–23] of nusinersen showed that the efficacy of the response varies widely across individual patients.

No patient in this program had previously received gene therapy, which may at least partly be related to all patients being older than 2 years: In contrast to the FDA, the EMA did not limit the use of onasemnogene abeparvovec to children of ≤ 2 years of age, along with the provision of dosing recommendations for children with a bodyweight of up to 21 kg by Novartis. To date, established safety and efficacy data are only available for patients less than 6 months of age; limited post-approval data on patients of up to 2 years of age and with a bodyweight of up to 13.5 kg have also been published. Therefore, European and German consensus statements recommend to particularly carefully consider the benefit/risk ratio in patients older than 6 months and express safety concerns, e.g. regarding hepatic and cardiac toxicities, for bodyweight-based doses of patients with ≥ 13.5 kg [24, 25].

Generally, the safety data observed in this CUP were in line with the safety profile of risdiplam in ongoing clinical studies. A pooled safety analysis from the ongoing clinical trials FIREFISH, SUNFISH, JEWELFISH and RAINBOWFISH, based on 465 patients and a total risdiplam exposure of 480.9 patient years, reported 709 AEs in patients with SMA1 (n = 77, AE and SAE rate of 441.80 and 70.41 per 100 patient years, respectively) and 3,244 AEs in patients with SMA2 (n = 388, AE and SAE rate of 65.67 and 21.10 per 100 patient years, respectively) [26]: Overall, headache, fever, and upper respiratory tract infections were the most common AEs. Diarrhea, nausea, rash, and headache were the most frequently observed risdiplam-related AEs, and pneumonia was the most common SAE in both SMA1 and SMA2. Most reported AEs and SAEs were associated with the underlying disease or disease progression, and the overall rate of AEs decreased over time with continued risdiplam treatment. No treatment-related safety findings led to withdrawal of risdiplam therapy.

An expanded access program (EAP) in the US with 73 patients with SMA1 and 82 patients with SMA2 also reported a similar safety profile for risdiplam as seen in pivotal clinical trials and no new safety signals [27].

Despite the limitations of a CUP-based data collection, our observations also indicated no new safety signals under real-world conditions. The on-label patient population will additionally comprise younger patients, patients who are eligible to receive nusinersen or onasemnogene abeparvovec, and patients with clinically diagnosed SMA3 or 1–4 *SMN2* gene copies.

Expanded access programs such as this CUP, whose primary purpose is to provide compassionate use-based access to unapproved therapies, are not designed for the systematic collection of data and do not intend to investigate scientific questions: CUPs do not show the rigor, control and close monitoring of clinical trials intending to generate data for the submission of drug applications [28]. As a result of these limitations, not all of the reported AEs could be linked to a distinct patient identity. Therefore endpoints such as time to first AE and number of events per participants were not captured, and potential associations of AEs with baseline characteristics were not analyzed. Our data are also limited because the CUP treatment plan and data collection were subject to the national regulatory framework (AMHV) that prevents CUPs from following certain requirements for clinical studies or even randomized controlled trials, e.g. measures to control selection and treatment bias. It is important to note that the collection of any effectiveness data and a longer follow-up period were not possible due to legal restrictions that limit data collection to documentation of the patients’ baseline eligibility and safety monitoring. Follow-up ended on June 14, 2021, due to termination of the CUP upon marketing authorization and after the subsequent wash-out phase. Despite all these limitations, real-world data and evidence such as the observations from this CUP and the US-based EAP are increasingly valued and should supplement the knowledge derived from clinical trials [28].

## Conclusion

Our observations from this CUP present the first real-world data of risdiplam in patients with SMA1 and SMA2 in Germany, with safety data generally in line with the safety profile derived from clinical trials. Real-world patient populations of both SMA1 and SMA2 comprise considerable numbers of patients without current SMA treatment, including children and adults of a wide age spectrum, who are not eligible for gene transfer therapy and who cannot tolerate or have failed treatment with nusinersen. We expect that recently approved oral risdiplam, which can be self-administered at home, has significantly reduced this treatment gap.

## Supplementary Information

**Additional file 1. Supplementary table S1**. Overview of key clinical trials.** Supplementary table S2**. List of SAEs by MedDRA System Organ Class and Preferred Term.** Supplementary table S3**. List of non-serious AEs by MedDRA System Organ Class and Preferred Term.

## Data Availability

The datasets generated and/or analyzed during this compassionate use program are not publicly available.
