# Correction to: Short-term safety results from compassionate use of risdiplam in patients with spinal muscular atrophy in Germany

**DOI:** 10.1186/s13023-022-02548-7

**Published:** 2022-10-25

**Authors:** Andreas Hahn, René Günther, Albert Ludolph, Oliver Schwartz, Regina Trollmann, Patrick Weydt, Markus Weiler, Kathrin Neuland, Martin Sebastian Schwaderer, Tim Hagenacker, the Risdiplam Compassionate Use Program Group

**Affiliations:** 1 Department of Child Neurology, University of Gießen, Giessen, Germany; 2 Department of Neurology, University Hospital Carl Gustav Carus, Technische Universität Dresden, Dresden, Germany; 3 Department of Neurology, University of Ulm, Ulm, Germany; 4 Department of Pediatric Neurology, Münster University Hospital, Münster, Germany; 5 Division of Pediatric Neurology, Department of Pediatrics, Friedrich-Alexander-Universität Erlangen-Nürnberg, Erlangen, Germany; 6 Department of Neurodegenerative Diseases and Gerontopsychiatry/Neurology, University of Bonn Medical Center, Bonn, Germany; 7 Department of Neurology, Heidelberg University Hospital, Heidelberg, Germany; 8 Roche Pharma AG, Grenzach-Wyhlen, Germany; 9 Department of Neurology, University Hospital Essen, Essen, Germany

## Correction to: Orphanet Journal of Rare Diseases (2022) 17:276 https://doi.org/10.1186/s13023-022-02420-8

Following publication of the original article [1], we have been notified that there is a typographical error in the Fig. 1 text. It should be as per below:

Figure 1: “111 patients received ≥ 1 risdiplam dose:”
