# Supplementary material for: Short-term safety results from compassionate use of risdiplam in patients with spinal muscular atrophy in Germany
Source: Orphanet J Rare Dis. 2022 Jul 19;17:276. doi: 10.1186/s13023-022-02420-8 (PMC9295446; doi:10.1186/s13023-022-02420-8)
Supplement: Supplementary file 1 — Additional file 1. Supplementary table S1. Overview of key clinical trials. Supplementary table S2. List of SAEs by MedDRA System Organ Class and Preferred Term. Supplementary table S3. List of non-serious AEs by MedDRA System Organ Class and Preferred Term. [file 13023_2022_2420_MOESM1_ESM.docx]

**Additional file 1**

**Supplementary table S1. Overview of key clinical trials.**

| **Trial** | **Design** | **Patients** | **Key efficacy results** |
| --- | --- | --- | --- |
| FIREFISH (NCT02913482) | Phase 2/3, open-label, multi-center trial; Part 1: dose-finding (n=21),  Part 2: safety and efficacy (n=41) | Patients with SMA1, age 1 – 7 months | - Primary endpoint met after 12 months: 12 infants (29%) were able to sit without support for at least 5 seconds (9) - At 24 months of treatment, 93% (38/41) of the participants were alive, further improvements in motor function were observed, and 83% (34/41) of infants did not require permanent ventilation (11) |
| SUNFISH (NCT02908685) | Phase 2/3, randomized, placebo-controlled, double-blind, multi-center trial; Part 1: dose-finding (n=51), Part 2: safety and efficacy (n=180) | Patients with SMA2 and 3, age 2 – 25 years | - Primary endpoint met after 12 months: statistically significant difference in the change from baseline in MFM32 at month 12 between patients treated with risdiplam (n=120) and placebo (10) - At month 24, patients receiving risdiplam maintained or continued to improve motor function and independence in activities of daily living (29) |
| JEWELFISH (NCT03032172) | Phase 2, multi-center, open-label trial (n=180) | Pre-treated patients*, age 6 months to 60 years | - Sustained, >2-fold increase in median SMN protein levels versus baseline, consistent with treatment-naïve patients previous SMA treatments (30) |
| RAINBOWFISH (NCT03779334) | Phase 2, multi-center, open-label, single-arm trial (n=~25) | Infants with genetically diagnosed and pre-symptomatic SMA, from birth to age 6 weeks | - Infants treated with risdiplam for at least 12 months (n=5) achieved motor milestones and reached the maximum score of 64 on the CHOP-INTEND (31) |

Key clinical trials to evaluate the safety, pharmacokinetics, pharmacodynamics and efficacy of risdiplam in different patient populations.

* Previously treated with RG7800, nusinersen, olesoxime or onasemnogene abeparvovec. CHOP-INTEND; Children's Hospital of Philadelphia Infant Test of Neuromuscular Disorders; MFM32, 32-item Motor Function Measure total score; SMA1, spinal muscular atrophy type 1; SMA2, spinal muscular atrophy type 2; SMN, survival motor neuron.

**Supplementary table S2.** **List of SAEs by MedDRA System Organ Class and Preferred Term.**

| **Serious adverse events, n (%)*** | | **SMA1** | **SMA2** | **Total** |
| --- | --- | --- | --- | --- |
| **Gastrointestinal disorders** | | - | 5 (5.0) | 5 (3.8) |
|  | **Abdominal pain** | - | 1 (1.0) | 1 (0.8) |
|  | **Constipation** | - | 1 (1.0) | 1 (0.8) |
|  | **Diarrhea** | - | 1 (1.0) | 1 (0.8) |
|  | **Large intestine perforation** | - | 1 (1.0) | 1 (0.8) |
|  | **Esophageal hypomotility** | - | 1 (1.0) | 1 (0.8) |
| **General disorders and  administration site conditions** | | - | 1 (1.0) | 1 (0.8) |
|  | **Pyrexia** | - | 1 (1.0) | 1 (0.8) |
| **Infections and infestations** | | 2 (6.7) | 2 (2.0) | 4 (3.0) |
|  | **Diverticulitis** | - | 2 (2.0) | 2 (1.5) |
|  | **Pneumonia** | 2 (6.7) | - | 2 (1.5) |

Of 111 patients who received at least one dose of risdiplam (31 patients with SMA1 and 80 patients with SMA2), 3 patients experienced at least 1 SAE (1 patient with SMA1 and 2 patients with SMA2). *Percentages are based on total number of AEs (30 in SMA1 and 100 in SMA2). MedDRA, Medical Dictionary for Regulatory Activities; SAE, serious adverse event; SMA1, spinal muscular atrophy type 1; SMA2, spinal muscular atrophy type 2.

**Supplementary table S3. List of non-serious AEs by MedDRA System Organ Class and Preferred Term.**

| **Non-serious adverse events, n (%)*** | | | **SMA1** | | **SMA2** | | **Total** | |
| --- | --- | --- | --- | --- | --- | --- | --- | --- |
| **Ear and labyrinth disorders** | | | | | | | | |
|  | Ear pain | | - | | 1 (1.0) | | 1 (0.8) | |
| **Gastrointestinal disorders** | | | | | | | | |
|  | Abdominal pain | | 2 (6.7) | | 2 (2.0) | | 4 (3.1) | |
|  | Abdominal pain lower | | 1 (3.3) | | - | | 1 (0.8) | |
|  | Abdominal pain upper | | - | | 1 (1.0) | | 1 (0.8) | |
|  | Aphthous ulcer | | - | | 4 (4.0) | | 4 (3.1) | |
|  | Cheilitis | | 1 (3.3) | | - | | 1 (0.8) | |
|  | Constipation | | - | | 4 (4.0) | | 4 (3.1) | |
|  | Diarrhea | | 3 (10.0) | | 9 (9.0) | | 12 (9.2) | |
|  | Dyspepsia | | - | | 1 (1.0) | | 1 (0.8) | |
|  | Dysphagia | | - | | 1 (1.0) | | 1 (0.8) | |
|  | Flatulence | | 1 (3.3) | | 1 (1.0) | | 2 (1.5) | |
|  | Gingival bleeding | | - | | 1 (1.0) | | 1 (0.8) | |
|  | Gingival pain | | 1 (3.3) | | 1 (1.0) | | 2 (1.5) | |
|  | Hematochezia | | - | | 1 (1.0) | | 1 (0.8) | |
|  | Hypesthesia oral | | - | | 1 (1.0) | | 1 (0.8) | |
|  | Nausea | | - | | 4 (4.0) | | 4 (3.1) | |
|  | Oral mucosal blistering | | - | | 1 (1.0) | | 1 (0.8) | |
|  | Tongue blistering | | - | | 1 (1.0) | | 1 (0.8) | |
|  | Tongue ulceration | | - | | 1 (1.0) | | 1 (0.8) | |
|  | Salivary hypersecretion | | 2 (6.7) | | - | | 2 (1.5) | |
|  | Vomiting | | - | | 1 (1.0) | | 1 (0.8) | |
| **General disorders and administration site conditions** | | | | | | | | |
|  | Asthenia | | - | | 1 (1.0) | | 1 (0.8) | |
|  | Fatigue | | - | | 1 (1.0) | | 1 (0.8) | |
|  | No adverse event** | | 1 (3.3) | | 3 (3.0) | | 4 (3.1) | |
|  | Pyrexia | | - | | 3 (3.0) | | 3 (2.3) | |
| **Infections and infestations** | | | | | | | |  |
|  | \| Fungal infection \| \| --- \| | - | | 1 (1.0) | | 1 (0.8) | |  |
|  | Nasopharyngitis | 1 (3.3) | | 1 (1.0) | | 2 (1.5) | |  |
|  | Respiratory tract infection viral | 1 (3.3) | | - | | 1 (0.8) | |  |
|  | Upper respiratory tract infection | - | | 1 (1.0) | | 1 (0.8) | |  |
| **Injury, poisoning and procedural complications** | | | | | | | |  |
|  | Circumstance or information capable of leading to medication error | 1 (3.3) | | 4 (4.0) | | 5 (3.8) | |  |
|  | Product preparation error | - | | 1 (1.0) | | 1 (0.8) | |  |
|  | Product use issue | 1 (3.3) | | - | | 1 (0.8) | |  |
|  | Skin pressure mark | 1 (3.3) | | - | | 1 (0.8) | |  |
|  | Underdose | - | | 2 (2.0) | | 2 (1.5) | |  |
|  | Wrong technique in product usage process | 1 (3.3) | | - | | 1 (0.8) | |  |
| **Investigations** | | | | | | | |  |
|  | Bleeding time prolonged | - | | 1 (1.0) | | 1 (0.8) | |  |
|  | Inflammatory marker increased | - | | 1 (1.0) | | 1 (0.8) | |  |
| **Metabolism and nutrition disorders** | | | | | | | |  |
|  | Decreased appetite | 1 (3.3) | | - | | 1 (0.8) | |  |
|  | Increased appetite | - | | 1 (1.0) | | 1 (0.8) | |  |
| **Musculoskeletal and connective tissue disorders** | | | | | | | |  |
|  | Arthralgia | - | | 2 (2.0) | | 2 (1.5) | |  |
|  | Arthropathy | - | | 1 (1.0) | | 1 (0.8) | |  |
|  | Back pain | - | | 1 (1.0) | | 1 (0.8) | |  |
|  | Bursitis | - | | 1 (1.0) | | 1 (0.8) | |  |
|  | Myalgia | - | | 1 (1.0) | | 1 (0.8) | |  |
|  | Myosclerosis | 1 (3.3) | | - | | 1 (0.8) | |  |
|  | Neck pain | - | | 1 (1.0) | | 1 (0.8) | |  |
|  | Pain in extremity | - | | 1 (1.0) | | 1 (0.8) | |  |
| **Neoplasms benign, malignant and unspecified (incl. cysts and polyps)** | | | | | | | |  |
|  | Lipoma | 1 (3.3) | | - | | 1 (0.8) | |  |
| **Nervous system disorders** | | | | | | | |  |
|  | Dizziness | - | | 1 (1.0) | | 1 (0.8) | |  |
|  | Headache | 1 (3.3) | | 6 (6.0) | | 7 (5.4) | |  |
|  | Hypesthesia | - | | 1 (1.0) | | 1 (0.8) | |  |
|  | Paresthesia | - | | 1 (1.0) | | 1 (0.8) | |  |
|  | Tremor | - | | 1 (1.0) | | 1 (0.8) | |  |

| **Product issues** | | | | |
| --- | --- | --- | --- | --- |
|  | Device defective | - | 1 (1.0) | 1 (0.8) |
|  | Device issue | - | 1 (1.0) | 1 (0.8) |
|  | Product packaging quantity issue | - | 1 (1.0) | 1 (0.8) |
|  | Syringe issue | - | 3 (3.0) | 3 (2.3) |
| **Psychiatric disorders** | | | | |
|  | Middle insomnia | 1 (3.3) | - | 1 (0.8) |
| **Renal and urinary disorders** | | | | |
|  | Bladder discomfort | 1 (3.3) | - | 1 (0.8) |
|  | Dysuria | 1 (3.3) | 1 (1.0) | 2 (1.5) |
|  | Pollakisuria | - | 1 (1.0) | 1 (0.8) |
| **Reproductive system and breast disorders** | | | | |
|  | Amenorrhea | - | 2 (2.0) | 2 (1.5) |
|  | Menstruation irregular | - | 1 (1.0) | 1 (0.8) |
|  | Vulvovaginal dryness | - | 1 (1.0) | 1 (0.8) |
| **Respiratory, thoracic and mediastinal disorders** | | | | |
|  | Cough | 1 (3.3) | 1 (1.0) | 2 (1.5) |
| **Skin and subcutaneous tissue disorders** | | | | |
|  | Acne | - | 1 (1.0) | 1 (0.8) |
|  | Erythema | 1 (3.3) | - | 1 (0.8) |
|  | Photosensitivity reaction | - | 1 (1.0) | 1 (0.8) |
|  | Rash | - | 1 (1.0) | 1 (0.8) |
|  | Skin lesion | - | 1 (1.0) | 1 (0.8) |
|  | Urticaria | 1 (3.3) | - | 1 (0.8) |

AEs sorted by MedDRA System Organ Class categories and Preferred Term. Of 111 patients who received at least one dose of risdiplam (31 patients with SMA1 and 80 patients with SMA2), 44 cases (13 cases with SMA1 and 31 cases with SMA2) were reported. *Percentages are based on total number of AEs (30 in SMA1 and 100 in SMA2). ** “No adverse event” refers to a special situation such as e.g. an administration error without any AE. AE, adverse event; MedDRA, Medical Dictionary for Regulatory Activities; SMA1, spinal muscular atrophy type 1; SMA2, spinal muscular atrophy type 2.
